# Irradiation and Alterations in Hippocampal DNA Methylation

**DOI:** 10.3390/epigenomes8030027

**Published:** 2024-07-05

**Authors:** Soren Impey, Jacob Raber

**Affiliations:** 1 Dow Neurobiology Laboratories, Legacy Research Institute Legacy Health Systems, 1225 NE 2nd Ave, Portland, OR 97232, USA; 2 Departments of Behavioral Neuroscience, Neurology, and Radiation Medicine, Division of Neuroscience, ONPRC, Oregon Health & Science University, 3181 SW Sam Jackson Park Road, Portland, OR 97239, USA

**Keywords:** radiation, DNA methylation, hippocampus, cognition, synaptic signature

## Abstract

The response of the brain to radiation is important for cancer patients receiving whole or partial brain irradiation or total body irradiation, those exposed to irradiation as part of a nuclear accident or a nuclear war or terrorism event, and for astronauts during and following space missions. The mechanisms mediating the effects of irradiation on the hippocampus might be associated with alterations in hippocampal DNA methylation. Changes in cytosine methylation involving the addition of a methyl group to cytosine (5 mC) and especially those involving the addition of a hydroxy group to 5 mC (hydroxymethylcytosine or 5 hmC) play a key role in regulating the expression of genes required for hippocampal function. In this review article, we will discuss the effects of radiation on hippocampal DNA methylation and whether these effects are associated with hippocampus-dependent cognitive measures and molecular measures in the hippocampus involved in cognitive measures. We will also discuss whether the radiation-induced changes in hippocampal DNA methylation show an overlap across different doses of heavy ion irradiation and across irradiation with different ions. We will also discuss whether the DNA methylation changes show a tissue-dependent response.

## 1. Introduction

**Figure 1:**
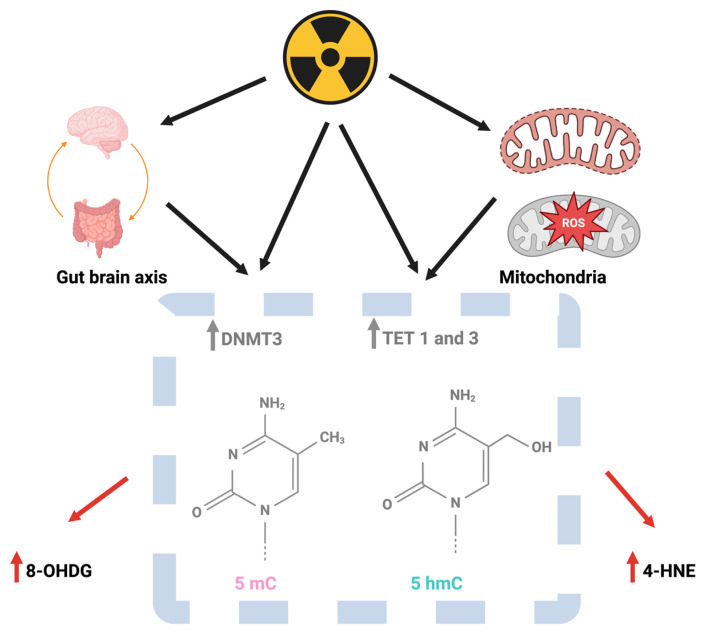
Following exposure to ionizing irradiation as part of cancer treatment, a nuclear attack or incident at a power plant, or space missions, effects on DNA methylation in and outside the brain might involve the bi-directional gut–brain axis and reactive oxygen species (ROS) in mitochondria. Irradiation has been shown to increase the hippocampal levels of the DNMT3, TET 1, and TET 3 enzymes involved in DNA methylation. Alterations in levels of 5 mC and 5 hmC, in turn, might be related to the increased levels of the injury markers 8-OHDG and 4-HNE observed following irradiation. Figure was generated using Biorender.com software.

Humans are exposed to ionizing irradiation as part of cancer treatment, a nuclear attack or incident at a power plant, or space missions (Figure 1). While historically, lower radiation doses and lower energies were used for cancer radiotherapy, recently, there has been an increased interest in considering ultra-high dose (40–300 Gy/s) radiation (UHDR) and higher energies for tumor control in cancer treatment. UHDR aims for less normal tissue injury than conventional therapy [1]. There is increased interest in the use of some of the radiation exposures found in the space environment for cancer therapy. For example, proton [2], helium [3], and carbon [4] radiotherapy are examples of irradiations that are being used and/or considered for use instead of conventional photon or gamma irradiation for improved cancer treatment. Proton therapy allows for delivering a higher dose to the tumor than the surrounding healthy tissue [5]. Carbon therapy is also being considered for rare and hard-to-treat tumors [4]. Compared to protons and carbon ions, helium ions offer intermediate physical and biological properties [6], higher relative biological effectiveness and dose-weighted linear energy transfer (LET_d_) [7], and improved lung sparing compared to proton therapy in patients with left-sided breast cancer therapy [8]. However, while these newer radiation therapies might be better with regard to tumor control, that does not mean that they are necessarily associated with less detrimental effects on the brain or outside the brain. For example, cognitive injury was comparable in UHDR and conventionally irradiated mice in a recent study, and only a difference was seen in one immune cell population in the hippocampus [9]. However, some studies revealed reduced cognitive injury using UHDR [10,11] or reduced cognitive injury and behavioral alterations at only distinct doses or only in female mice [12,13]. With regard to simulated space irradiation, behavioral and cognitive data and various compensatory and/or injury pathways have been reported involving unusual dose-response curves (for a review, see [14]). With regard to the pathway changes, reduced neurogenesis has been observed at relatively higher radiation doses (5 or 10 Gy of photon or gamma irradiation [15,16] and 1 Gy of heavy ion irradiation [17,18]). Morphological changes, including changes in synaptic density, number and density of spines, measures of synaptic function, hippocampal network stability, vascular effects, DNA damage and oxidative stress, and neuroinflammation, have also been reported following heavy ion and conventional irradiation [19,20,21,22,23,24,25]. Effects on the brain following irradiation might involve peripheral immune responses, including infiltrating neutrophils [9], the gut microbiome, and the bi-directional gut–brain axis [26,27]. Increasing evidence supports that X-ray and gamma irradiation are associated with epigenetic changes, including alterations in DNA methylation involving Long Interspersed Nucleotide Element 1 (LINE-1) and *Alu* elements [28]. Alterations in DNA methylation involving these genetic elements are also seen following simulated space radiation [29,30,31,32,33,34,35]. In addition to these targeted analyses of DNA methylation, unbiased analyses have also revealed alterations in DNA methylation following simulated space radiation, involving protons [36,37,38,39], ^56^Fe ions [37,40], or ^28^Si ions [39]. In this review, we discuss: (1) the effects of radiation on hippocampal DNA methylation; (2) whether these effects are associated with hippocampus-dependent cognitive measures and molecular measures in the hippocampus involved in cognitive measures; (3) whether the radiation-induced changes in hippocampal DNA methylation show an overlap across different doses of heavy ion irradiation and across irradiation with different ions; and (4) whether the DNA methylation changes show a tissue-dependent response.

## 2. Methylation and Demethylation—Modifications and Enzymes

**Figure 2:**
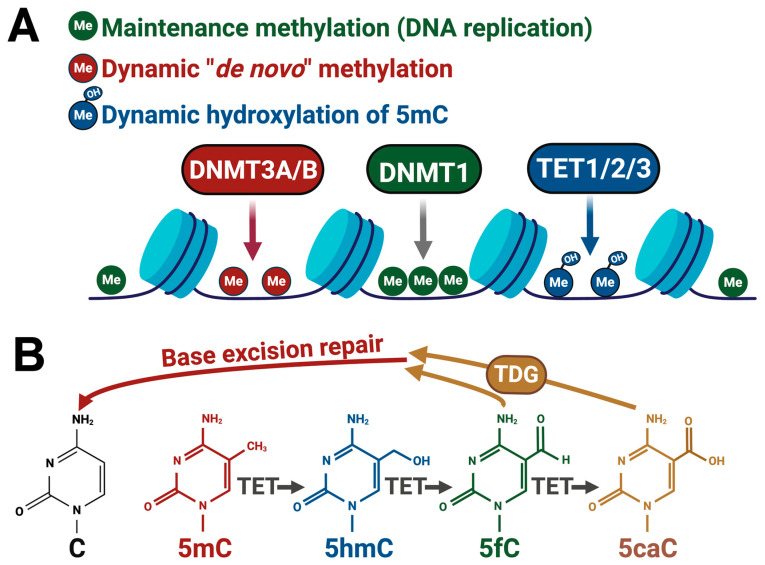
Methylation and demethylation mechanisms. (**A**) Diagram depicts methyltransferase enzymes and their functional roles, as well as the TET family of methyl-hydroxylases. (**B**) TET-family enzymes actively demethylate 5mC via step-wise oxidation followed by TDG-mediated glycosylation and base-excision repair of the abasic site to cytosine. Figure was generated using Biorender.com software.

DNA methylation of the C5 position of cytosine generates 5-methyl-cytosine (5mC), predominantly occurring at cytosines, followed by a guanine (CpG) [41,42,43] (Figure 2). Most tissues appear to be devoid of non-CpG methylation, but cytosine methylation adjacent to other bases (CpH) is found at modest levels in the central nervous system [43,44]. DNA methylation is catalyzed by DNMT1, DNMT3A, and DNMT3B, with DNMT1 largely functioning as a “maintenance” methylase of hemimethylated DNA during replication and the two DNMT3s functioning as de novo methylases responsible for the dynamic methylation of CpGs and CpHs [45]. Methylation can be spontaneously lost during DNA replication or repair and by deamination into thymidine. 5mC can also be actively oxidized by the Tet family of enzymes to a new relatively stable base, 5-hydroxymethyl-cytosine (5hmC), that is found at high levels in the brain [46,47,48]. The Tet family of enzymes can also actively demethylate 5hmC by sequentially oxidizing it to 5-formyl-cytosine and 5-carboxyl-cytosine bases, which can be removed via TDG-mediated base-excision repair [47,49,50].

## 3. Potential Mechanisms by Which Radiation Exposure Regulates DNA Methylation Enzymes

Some studies support that DNA methylation enzymes in the hippocampus, including DNA methyltransferase (DNMT)3 and ten-eleven translocation methylcytosine dioxygenase (TET) enzymes 1 and 3, are increased 2 h and 24 h following ^28^Si ion irradiation [51]. In addition, CA1 hippocampal TET2 levels are reduced by two, but not twenty, weeks following ^56^Fe ion irradiation [39]. It is unclear whether these distinct patterns are due to differences in the type of irradiation, the time interval between irradiation and analysis, or potential differential effects on the distinct DNA methylation enzymes. In physicians with work-related exposure to irradiation, DNMT levels in peripheral blood leukocytes are increased and associated with increased levels of markers of oxidative damage, 8-hydroxy-2′-deoxyguanosine (8-OHDG) and 4-hydroxynonenal (4-HNE), and the total cumulative personal dose equivalent is positively correlated with levels of Dnmts, 8-OHDG, and 4-HNE [39]. Total DNA methylation levels are not altered, a result consistent with the lack of alterations in global DNA methylation levels following irradiation in preclinical studies.

Mitochondria might play an important role in mediating DNA methylation following radiation exposure. Radiation exposure impacts mitochondrial function, and impaired mitochondrial function modulates epigenetic regulation [39]. This might relate to the reactive oxygen species in the mitochondria and related damage in mitochondrial DNA [52,53]. Consistent with an important role for mitochondria in mediating DNA methylation following radiation exposure, increased levels of enzymes and compounds involved in regulating anti-oxidant levels show radioprotective properties [54,55,56,57,58].

**Figure 3:**
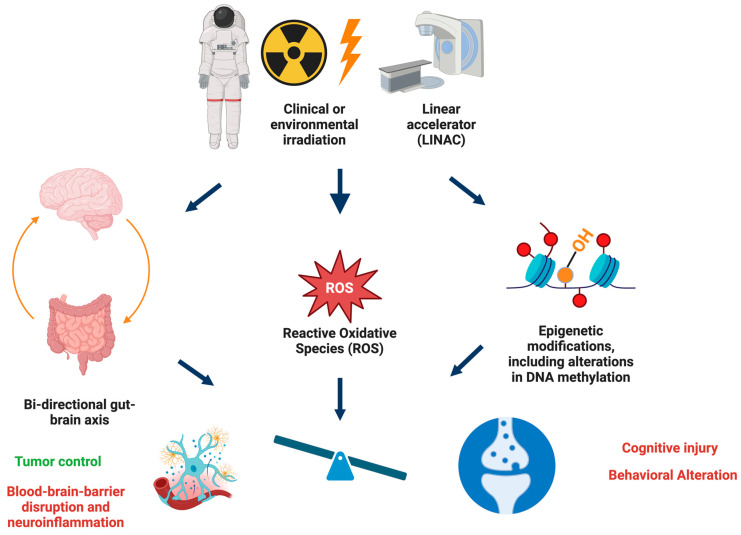
Radiation studies in cancer patients and in preclinical models have highlighted that therapeutic strategies to control the tumor are often associated with behavioral alterations and cognitive injury, involving detrimental effects on synaptic function. It is recognized that simulated space irradiation is not only pertinent to astronauts during and following missions. There is increasing interest in the use of radiation as part of the space environment in cancer therapy, including the use of protons and carbon in cancer patients. Detrimental effects on the blood–brain barrier and neuroinflammation might be a large contributor to these detrimental effects as well. These detrimental effects are not limited to radiation therapy and are often seen following chemotherapy and immunotherapy as well. The effects of radiation on the brain, including those of often-used clinical radiation and simulated space irradiation, are associated with epigenetic alterations. The gut–brain axis and ROS might play a role in these pathways. Figure was generated using Biorender.com software.

In addition to mitochondria, the gut microbiome, including microbe-derived metabolites and the gut–liver–brain axis, might play an important role in regulating DNA methylation following radiation exposure [59], especially as the gut microbiome is affected by radiation exposure [26,27,60]. In the context of tumor control in cancer patients, the same pathways required to control the tumor often negatively affect behavioral and cognitive performance. Epigenetic studies indicate that in the brain, synaptic injury might be especially vulnerable to radiation-induced detrimental effects (Figure 3).

## 4. Regulation of Gene Expression

Between 80 and 90% of genomic CpG cytosines are methylated, but the majority of unmethylated cytosines are concentrated in CpG islands that are co-located with the promoters of ~50% of mammalian genes [41,61]. Analyses of genome-wide transcription show that demethylated promoter-proximal CpG islands tend to be active, suggesting a link between methylation and transcription [42,61]. The CXXC protein CFP1 binds tightly to unmethylated CpGs and recruits H3K4 methyltransferases, and the H3K4 methylatransferase MLL has also been shown to protect CpG from methylation [61]. Interestingly, DNMT3 methyltransferase activity is inhibited by methylated H3K4, suggesting redundant mechanisms for the protection of CpGs from methylation. 5hmC, TET-family enzymes, and TDG are all enriched in transcribed regions and adjacent to some promoters, suggesting a role for TET-family members in promoter demethylation [50,62,63]. TET1 and TDG also interact with GADD45a, which in turn is recruited by R-loops—a DNA-RNA structure that forms at actively transcribed genes [64,65]. More recently, it was shown that 5-formyl-cytosine is enriched at enhancers and that TDG binds to and enhances CBP/P300-mediated transcription, raising the possibility that TET-mediated demethylation enhances CBP/P300-associated transcription via enhancer interactions [65,66,67].

**Figure 4:**
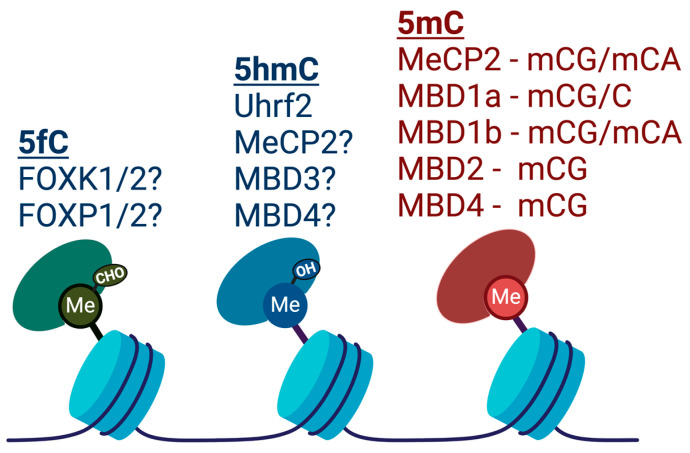
Diagram depicting proteins that bind to methylated DNA, sorted by the type of methylation. A “?” denotes conflicting evidence or a lack of validation by other studies. For 5mC, we have also indicated whether the protein interacts with CpG or CpA sites. Figure was generated using Biorender.com software.

DNA methylation at repressed genes is thought to be a result of the recruitment of DNMT3 via interactions with H3K9 methyltransferase corepressor complexes that are, in turn, recruited by repressive transcription factors [67]. Methyl-CpG binding proteins, such as MeCP2, interact with and recruit corepressor complexes (e.g., SIN3A and NuRD) to methylated regions [68] and have also been shown to directly repress transcription of methylated templates in vitro [42]. In particular, the Rett syndrome-associate gene, MeCP2, binds to methylated CpG and CpA stretches [69] and has recently been shown to attenuate the transcription initiation of longer genes (e.g., >100 kb) [70]. A number of proteins have been shown to interact with 5mC, 5hmC, or 5fC [71,72], but many of these await structural and functional validation by other studies [71] (Figure 4). Uhrf2 has been shown to interact with 5hmC and facilitate TdG-mediated demethylation [73]. MeCP2, MBD3, and MBD4 have also been reported to interact with 5hmC, but other studies have been unable to confirm this finding [71].

## 5. Cognitive Tests Typically Used in Radiation Studies

**Figure 5:**
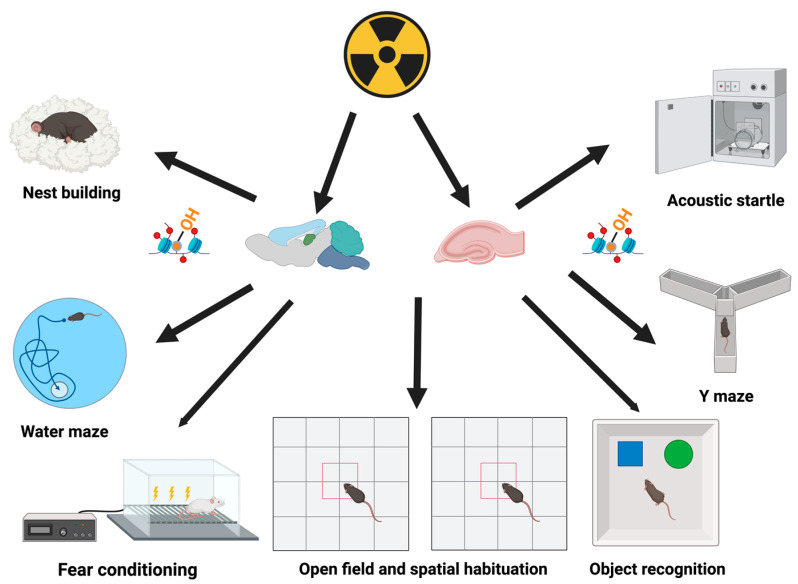
Behavioral and cognitive tests are often used to assess the effects of irradiation on the brain. Historically, the hippocampus has been the focus of studies assessing the effects of irradiation on the brain. For example, neurogenesis has been studied, and based on the susceptibility of the hippocampus to radiation effects, hippocampal sparing is being considered in radiation therapy. However, it is recognized that the effects of irradiation are seen at doses below those affecting neurogenesis. The quality of nest building is considered a measure related to the activities of daily life for humans. In the water maze, spatial navigation to a visible or hidden platform or spatial memory retention in a probe trial (no platform) is assessed. Fear learning and memory can be assessed in the fear conditioning test. Depending on the design, either hippocampus-dependent contextual and/or hippocampus-independent cued fear conditioning is assessed. In the acoustic startle response test, the response to an acoustic stimulus, sometimes in the presence of another aversive stimulus, is assessed. In the Y maze, hippocampus-dependent spatial alternation is assessed in a single trial. In the spatial Y maze version of this test, there are two trials. In the first trial, access to one arm is blocked. Following a delay, access to the originally blocked arm is made available, and visits to that arm and entries into that arm are analyzed. Alterations in behavioral and cognitive performance following irradiation are associated with epigenetic changes in pertinent brain regions. These epigenetic effects are not limited to the brain and are also seen in other tissues following irradiation. Figure was generated using Biorender.com software.

The following cognitive tests are typically used in radiation studies: (1) nest building; (2) spatial habituation learning by exposing animals repeatedly to the same environment; (3) object recognition; (4) fear learning and memory; (5) analysis of hippocampal networks by exposing animals twice to the same environment or different environments; (6) spontaneous alternation in the Y maze; (7) spatial learning and memory in the water maze; (8) the acoustic startle response; and (9) fear conditioning (Figure 5).

## 6. Effects of Radiation on Hippocampal DNA Methylation and Cognitive Measures: Association between Cognitive Injury, Hippocampal Networks, Brain Injury Biomarkers, and DNA Methylation 

### 6.1. Cognitive Injury

Radiation exposure, as part of cancer treatment [74,75,76,77,78] or environmental radiation exposure [79,80], can affect the brain. For example, occupational chronic ionizing radiation exposure increased the risk of PD in workers at a nuclear facility [81]. Historically, hippocampal injury [82] and, as a result, hippocampal sparing [83] during radiotherapy have been driven by the susceptibility of neuroprogenitor cells in the subventricular and subgranular neurogenic zones to radiation exposure [18,25,84,85,86]. Several radiation studies support an association of radiation-induced cognitive injury with altered DNA methylation [37,39,40,51]. A limitation of these association analyses is that they do not show causality and that alterations in DNA methylation might reflect beneficial compensatory pathway changes as well as injury pathways. Compounds that modulate DNA methylation are typically not specific to specific genes or pathways and often have off-target effects. Also, based on the complexity and the number of genes that show altered DNA methylation patterns following radiation exposure, it is hard to simulate these patterns in the absence of radiation exposure or specifically block them in the presence of radiation exposure. However, there are trans-generational epigenetic effects that allow simulating epigenetic radiation signatures in the absence of direct radiation exposure [87,88,89,90,91,92,93,94]. For example, paternal irradiation in mice significantly affects the expression of genes involved in rhythmic processes, including genes involved in DNA-dependent transcriptional regulation, in their first-generation offspring, and this likely involves alterations in DNA methylation [95]. However, as this study involved first-generation offspring, possible alterations in paternal or maternal care that might have contributed to the epigenetic changes in the offspring cannot be excluded.

### 6.2. DNA Methylation Changes Show a Tissue-Dependent Response; Neurodegenerative Pathways

While high radiation doses, such as those used as part of cancer therapy, can cause severe tissue destruction, lower doses, such as those present in the space environment astronauts are exposed to during missions, can induce cognitive impairments without signs of overt tissue damage. As indicated earlier, morphological changes, including changes in synaptic structure and function, hippocampal network stability, vascular effects, DNA damage and oxidative stress, and neuroinflammation, have been reported following heavy ion and conventional irradiation [19,20,21,22,23,24,25]. Consistent with this pattern, ^56^Fe and ^28^Si ion simulated space radiation-induced alterations in hippocampal DNA methylation revealed enriched pathways for oxidative phosphorylation and pathways associated with several neurodegenerative diseases, including Huntington’s disease, Alzheimer’s disease, and Parkinson’s disease [37,39]. Oxidative stress has been linked to the neuropathology of these neurodegenerative disorders [96], suggesting that a common theme in radiation-induced brain injury might involve persistent oxidative stress that, in turn, negatively affects synaptic function and triggers pathways associated with neurodegenerative disorders. 

The unbiased nature of the analysis of hippocampal DNA methylation also allows for assessing which pathways anticipated to be altered following radiation might not be. In our short (2-week) and long (20-week) mouse radiation exposures involving protons [37,38], ^56^Fe ions [37,40], and ^28^Si ions [39], we did not detect alterations in immune pathways. This is remarkable, as targeted/biased analysis of radiation-induced injury revealed activation of microglia and specific cytokines, chemokines, and other immune markers [97,98,99,100]. It is conceivable that alterations in immune pathways were not revealed as our analyses involve whole brain regions or the whole left ventricle of the heart following radiation exposure and not a single cell level of analysis. For example, following cranial UHDR or conventional irradiation, there was an alteration in the number of infiltrating immune cells in the hippocampus revealed by flow cytometry [9], and it is conceivable that if only a minority of tissue cells show an immune signature, this might only be revealed by a single-cell level of analysis.

## 7. Genome-Wide Methylation Analyses

**Figure 6:**
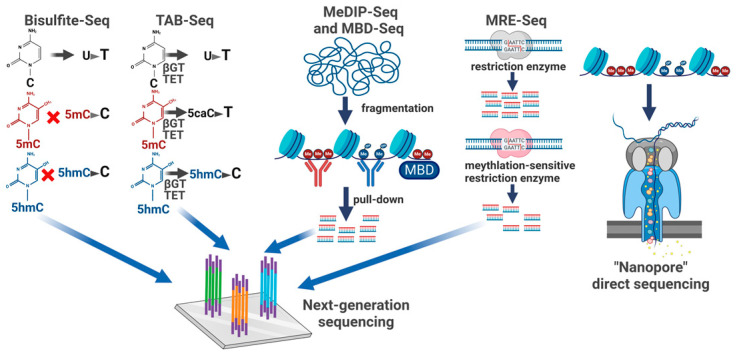
Methodologies for genome-wide profiling of cytosine methylation. Bisulfite converts non-methylated bases to uracil, which is then converted to thymidine via library amplification. Conventional bisulfite-Seq fails to distinguish 5mC from 5hmC. TAB-Seq and other related approaches use β glucosyltransferase (βGT) to protect 5hmC, followed by enzymatic or chemical conversion of non-protected bases. In the case of TAB-Seq, TET is used to sequentially oxidize 5mC, followed by bisulfite treatment, which converts the resulting 5caC or 5fC to uracil but spares the protected 5hmC. MeDIP-Seq and MBD-Seq use antibodies, or methyl-binding domains, to pull down methylated chromatin fragments. MeDIP-Seq can interrogate 5mC, 5hmC, 5fc, or 5caC through the use of specific antibodies. MRE-Seq digests DNA with restriction enzymes that cut specific CpG sequences or are blocked by methylation of these CpG sequences. Sites that are resistant to digestion are scored as methylated. Recently, “nanopore” sequencing of DNA has been used to create genome-wide maps of 5mC and 5hmC at single-nucleotide resolution. Figure was generated using Biorender.com software.

The earliest method for genome-wide methylation analysis was the use of methylation-blocked restriction enzymes to profile the absence of methylation at a single nucleotide resolution (MRE-Seq). The disadvantages of this technique were the limited number of enzymes that recognize CpG-containing sequences as well as the requirement for complete digestion [101]. A subsequent approach used antibodies to 5mC or methyl-binding proteins to precipitate and enrich for methylated DNA (MeDIP-Seq and MBD-Seq), which was then subjected to next-generation sequencing or microarray analysis (Figure 6). Although these techniques can interrogate methylation on a global basis, their resolution is limited by the size of the DNA fragment (200–300 bp) and by the inability to adequately segment highly methylated regions. Similar pull-down approaches have also been used for 5hmC, 5caC, and 5fC, and DIP-Seq methods are widely used due to their low cost and high specificity [101]. Bisulfite base conversion approaches were first used to sequence genomes via the use of restriction enzymes that cut specific CpGs, but subsequent studies used whole-genome bisulfite sequencing (WGBS) to profile methylation [101]. The main limitation of these approaches is the loss of complexity due to the conversion of unmethylated “C” to “T” as well as the inability of bisulfite conversion to distinguish 5mC from 5hmC (in the brain, up to 20% of methylated cytosines are 5hmC) [101,102]. Subsequent conversion approaches have used enzymatic or chemical approaches to differentially modify and detect 5mC and 5hmC [102]. For example, TAB-Seq used bisulfite sequencing paired with TET-mediated complete oxidation of 5mC to distinguish β-glucosyltransferase-protected 5hmC from 5mC [103], while oxBS-Seq used potassium perrhenate to selectively oxidize 5hmC and distinguish it from 5mc [104]. A major limitation of these approaches is the requirement to conduct two rounds of WGBS, along with the coverage limitations of WGBS due to loss of complexity. Two recent approaches circumvented these limitations by employing β-glucosyltransferase modification of 5hmC and chemical treatments to selectively distinguish 5mc from 5hmC without a requirement for bisulfite sequencing [102,105,106]. More recently, nanopore sequencing has been used to directly identify 5mC and 5hmC via whole-genome sequencing and currently represents the gold standard in terms of sensitivity and coverage [107].

## 8. Integrated Analyses of Radiation Effects on Hippocampal Cognitive and Molecular Measures: Overlapping DNA Methylation Changes across Various Doses and Ion Types

While one can pursue DNA methylation analyses following a single type of radiation exposure, an important question to address is whether there might be a common signature. This common signature could serve as a biomarker of the hippocampal radiation response and facilitate the development and testing of mitigating strategies to reduce or even prevent cognitive injury following radiation exposure. Therefore, as part of our DNA methylation simulated radiation studies, we compared hippocampal DNA hydroxymethylation following proton, ^56^Fe ion, and ^28^Si ion irradiation [39] (see Table 1).

**Figure 7:**
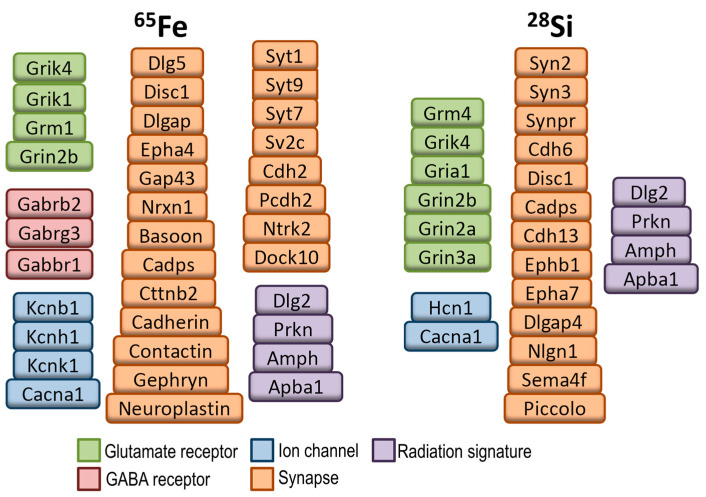
Selected genes from the “synapse” gene ontology were associated with increased 5hmC following ^65^Fe and ^28^Si irradiation of the mouse hippocampus. The genes highlighted in purple are radiation signature genes that were associated with increased 5hmc in response to a proton, ^65^Fe, or ^28^Si irradiation. Data were adapted and selected from [39].

Indeed, we identified a common signature involving 45 genes, with the glutamatergic synapse and postsynaptic density being mostly affected. We reported a highly significant overlap between ^28^Si, ^56^Fe, and proton DHR-associated genes, which included genes linked to radiation in other studies [108]. One of the 45 genes, PIAS1, was linked to irradiation in a database largely containing data from photon radiation studies [108]. Annotation of each of the 45 genes revealed that 23 of the 45 genes were associated with studies indicating a radiation response. For example, the gene Npas3 was reported to decrease following ^12^C ion irradiation [109]. The fact that genes were revealed following radiation exposures not involving ^12^C ions that were affected in separate ^12^C ion irradiation studies supports the power of identifying a common signature and might help with the plans to treat more cancer patients around the world with carbon. The common synaptic signatures revealed in our DNA methylation studies are consistent with changes in glutamatergic and other synaptic genes seen in our previous studies and in other simulated space radiation studies (see Figure 7) [20,110,111,112,113,114]. While we and others have focused a lot of radiation studies on the hippocampus, it will be important to expand these kinds of analyses to other brain regions to determine if similar pathways are affected across the brain or not. Regardless, these results support this approach and the potential to use a common signature to reduce or even prevent the detrimental effects of environmental and clinical radiation exposures.

## 9. Summary

The mechanisms mediating the effects of irradiation might be associated with alterations in hippocampal DNA methylation. These effects are associated with hippocampus-dependent cognitive injury and molecular measures in the hippocampus involved in cognitive measures. The DNA methylation changes following radiation exposure show a tissue-dependent response. Strikingly, the radiation-induced changes in hippocampal DNA methylation show an overlap across different doses of heavy ion irradiation and across irradiation with different ions and reveal a synaptic signature. This important result provides a valuable biomarker of cognitive injury following radiation exposure. This result also indicates that mitigating strategies targeting pathways and molecular targets as part of this overlap can be used to inhibit or even prevent cognitive injury following radiation exposures astronauts are exposed to during missions and patients are exposed to as part of cancer therapy.

## Figures and Tables

**Table 1:** Radiation qualities of DNA methylation studies.

Radiation Type	Energy	Dose (Gy)	Hippocampal Dissection Time Point
^56^Fe	600 MeV/n	0, 0.1, 0.2, and 0.4	5 and 23 weeks following irradiation or sham-irradiation
^28^Si	600 MeV/n	0, 0.3, 0.6, and 0.9	5 and 23 weeks following irradiation or sham-irradiation
Protons	150 MeV/n	1	5 and 23 weeks following irradiation or sham-irradiation

## Data Availability

The research data from the studies involving the authors were shared as part of the cited publications.
